# Supplementary material for: Dynamic gamma modulation of hippocampal place cells predominates development of theta sequences
Source: eLife. 2025 Apr 11;13:RP97334. doi: 10.7554/eLife.97334 (PMC11991698; doi:10.7554/eLife.97334)
Supplement: MDAR checklist [file elife-97334-mdarchecklist1.docx]

**Materials Design Analysis Reporting (MDAR)**

**Checklist for Authors**

**Materials:**

| **Newly created materials** | **Indicate where provided: section/figure legend** | **N/A** |
| --- | --- | --- |
| The manuscript includes a dedicated "materials availability statement" providing transparent disclosure about availability of newly created materials including details on how materials can be accessed and describing any restrictions on access. | Not currently checked |  |
|  |  |  |
| **Antibodies** | **Indicate where provided: section/figure legend** | **N/A** |
| For commercial reagents, provide supplier name, catalogue number and [RRID](https://scicrunch.org/resources), if available. | Not currently checked |  |
|  |  |  |
| **DNA and RNA sequences** | **Indicate where provided: section/figure legend** | **N/A** |
| Short novel DNA or RNA including primers, probes: Sequences should be included or deposited in a public repository. | Not currently checked |  |
|  |  |  |
| **Cell materials** | **Indicate where provided: section/figure legend** | **N/A** |
| Cell lines: Provide species information, strain. Provide accession number in repository OR supplier name, catalog number, clone number, OR RRID. | Not currently checked |  |
| Primary cultures: Provide species, strain, sex of origin, genetic modification status. | Not currently checked |  |
|  |  |  |
| **Experimental animals** | **Indicate where provided: section/figure legend** | **N/A** |
| Laboratory animals or Model organisms: Provide species, strain, sex, age, genetic modification status. Provide accession number in repository OR supplier name, catalog number, clone number, OR RRID. | 1-2 months male Long-Evans rat from Charles River Laboratories by Vitalriver company. |  |
| Animal observed in or captured from the field: Provide species, sex, and age where possible. | Not currently checked |  |
|  |  |  |
| **Plants and microbes** | **Indicate where provided: section/figure legend** | **N/A** |
| Plants: provide species and strain, ecotype and cultivar where relevant, unique accession number if available, and source (including location for collected wild specimens). | Not currently checked |  |
| Microbes: provide species and strain, unique accession number if available, and source. | Not currently checked |  |
|  |  |  |
| **Human research participants** | **Indicate where provided: section/figure legend) or state if these demographics were not collected** | **N/A** |
| If collected and within the bounds of privacy constraints report on age, sex, gender and ethnicity for all study participants. | Not currently checked |  |

**Design:**

| **Study protocol** | **Indicate where provided: section/figure legend** | **N/A** |
| --- | --- | --- |
| If the study protocol has been pre-registered, provide DOI. For clinical trials, provide the trial registration number OR cite DOI. | Not currently checked |  |
|  |  |  |
| **Laboratory protocol** | **Indicate where provided: section/figure legend** | **N/A** |
| Provide DOI OR other citation details if detailed step-by-step protocols are available. | Not currently checked |  |
|  |  |  |
| **Experimental study design (statistics details) *** | | |
| **For in vivo studies: State whether and how the following have been done** | **Indicate where provided: section/figure legend. If it could have been done, but was not, write “not done”** | **N/A** |
| Sample size determination | Not done |  |
| Randomisation | Not currently checked |  |
| Blinding | Not currently checked |  |
| Inclusion/exclusion criteria | Rats that weighed over 400g and were able to move normally in one direction on a circular track were included. |  |
|  |  |  |
| **Sample definition and in-laboratory replication** | **Indicate where provided: section/figure legend** | **N/A** |
| State number of times the experiment was replicated in the laboratory. | The experiment was repeated on each rat for 1-4 sessions |  |
| Define whether data describe technical or biological replicates. | Not currently checked |  |
|  |  |  |
| **Ethics** | **Indicate where provided: section/submission form** | **N/A** |
| Studies involving human participants: State details of authority granting ethics approval (IRB or equivalent committee(s), provide reference number for approval. | Not currently checked |  |
| Studies involving experimental animals: State details of authority granting ethics approval (IRB or equivalent committee(s), provide reference number for approval. | All experiments were conducted according to the guidelines of the Animal Care and Use Committee of Tianjin University (Approval No. TJUE-2023-142). |  |
| Studies involving specimen and field samples: State if relevant permits obtained, provide details of authority approving study; if none were required, explain why. | Not currently checked |  |
|  |  |  |
| **Dual Use Research of Concern (DURC)** | **Indicate where provided: section/submission form** | **N/A** |
| If study is subject to dual use research of concern regulations, state the authority granting approval and reference number for the regulatory approval. | Not currently checked |  |

**Analysis:**

| **Attrition** | **Indicate where provided: section/figure legend** | **N/A** |
| --- | --- | --- |
| Describe whether exclusion criteria were pre-established. Report if sample or data points were omitted from analysis. If yes, report if this was due to attrition or intentional exclusion and provide justification. | Not currently checked |  |
|  |  |  |
| **Statistics** | **Indicate where provided: section/figure legend** | **N/A** |
| Describe statistical tests used and justify choice of tests. | Normally distributed data were analyzed using paired two-sided t-tests (two groups), Student’s t test (two variables), One-sample t test (one variables), repeated ANOVA with Bonferroni post hoc analysis for multiple comparisons (≥3 groups).  Not normally distributed data were analyzed using Chi-squared test and Kolmogorov-Smirnov test (two variables). Very large sample size data were analyzed using Generalized linear mixed model is with Bonferroni post hoc analysis for multiple comparisons (≥3 groups). Circular data were analyzed using shown Watson-Williams multi-sample test and Mardia-Watson-Wheele multi-sample test. All data was show as mean ± sem. |  |
|  |  |  |
| **Data availability** | **Indicate where provided: section/submission form** | **N/A** |
| For newly created and reused datasets, the manuscript includes a data availability statement that provides details for access (or notes restrictions on access). | Not currently checked |  |
| When newly created datasets are publicly available, provide accession number in repository OR DOI and licensing details where available. | Not currently checked |  |
| If reused data is publicly available provide accession number in repository OR DOI, OR URL, OR citation. | Not currently checked |  |
|  |  |  |
| **Code availability** | **Indicate where provided: section/figure legend** | **N/A** |
| For any computer code/software/mathematical algorithms essential for replicating the main findings of the study, whether newly generated or re-used, the manuscript includes a data availability statement that provides details for access or notes restrictions. | Not currently checked |  |
| Where newly generated code is publicly available, provide accession number in repository, OR DOI OR URL and licensing details where available. State any restrictions on code availability or accessibility. | Custom scripts for analysis and visualization have been deposited in GitHub repositories (https://github.com/WNunc/Dynamic_gamma_modulation). |  |
| If reused code is publicly available provide accession number in repository OR DOI OR URL, OR citation. | Not currently checked |  |

**Reporting:**

The MDAR framework recommends adoption of discipline-specific guidelines, established and endorsed through community initiatives.

| **Adherence to community standards** | **Indicate where provided: section/figure legend** | **N/A** |
| --- | --- | --- |
| State if relevant guidelines (e.g., ICMJE, MIBBI, ARRIVE, STRANGE) have been followed, and whether a checklist (e.g., CONSORT, PRISMA, ARRIVE) is provided with the manuscript. | Not currently checked |  |
